# Transesophageal Ultrasound Guidance for Endovascular Interventions on the Aorta

**DOI:** 10.1055/s-0042-1743107

**Published:** 2022-05-31

**Authors:** Mireya Castro-Verdes, Xun Yuan, Andreas Mitsis, Wei Li, Christoph A. Nienaber

**Affiliations:** 1 Department of Echocardiography and Paediatric Cardiology, Royal Brompton and Harefield, National Health Service (NHS) Foundation Trust, London, United Kingdom; 2 Department of Cardiology and Aortic Centre, Royal Brompton & Harefield National Health Service Foundation Trust, London, United Kingdom; 3 National Heart and Lung Institute, Faculty of Medicine, Imperial College London, London, United Kingdom; 4 Department of Cardiology, Nicosia General Hospital, Strovolos, Cyprus; 5 Department of Echocardiography and Congenital Heart Disease, Royal Brompton & Harefield National Health Service Foundation Trust, London, United Kingdom

**Keywords:** transesophageal echocardiogram, aorta, percutaneous intervention, aortic dissection, thoracic endovascular aortic repair

## Abstract

Aortic pathologies in general require a multidisciplinary approach and decision-making to integrate elements of clinical acuity, vascular pathology, individual comorbidity, and risk assessment; thus, ideally it is a center with access to multiple imaging modalities and expertise in all treatment options. Besides classic open surgical options, endovascular procedures have been accepted for a variety of aortic pathologies. More recently, novel transcatheter interventions even to the proximal aorta have been introduced, particularly for patients unfit for open surgery. Nevertheless, the role of transesophageal echocardiography to guide percutaneous aortic interventions is not well established, notwithstanding the growing potential as an ancillary tool to guide the procedure and document success.

## Introduction

Thoracic endovascular aortic repair (TEVAR) has been accepted as an important therapeutic modality for treatment of acute and chronic aortic pathologies, such as acute aortic syndromes and chronic aneurysms of the descending aorta. 1 More recently, minimalistic interventions on the aorta have emerged offering new treatment options, particularly in highly selected patients with distinct pathologies considered at a prohibitively high surgical risk. 2 These include the use of coils and vascular plugs to seal residual entry points to perfused and expanding false lumens (FLs). With the evolution and acceptance of transcatheter structural heart procedures over the past 15 years, echocardiography has undoubtfully become important for procedural guidance. While there is consensus on the use of periprocedural echocardiography in structural cardiac interventions such as mitral valve interventions or left auricular appendage closure, ultrasound assistance is not yet widely accepted and standardized as an adjunct to interventional procedures on the aorta. This article intends to focus on the importance of periprocedural transesophageal ultrasound guidance in the context of endovascular aortic interventions.

## Echocardiographic Assessment of the Aorta

The advantages of ultrasound in the assessment of patients with aortic diseases, in comparison with other imaging modalities, are availability and cost-effectiveness, as well as mobility to be used at the bedside in unstable patients or in theater. 3 Moreover, ultrasound interrogation of the aorta should be an integral part of any standard echocardiogram. Even though transthoracic echocardiography (TTE) cannot provide a comprehensive assessment of the entire vessel, 4 it is useful to interrogate proximal ascending aortic segments, the aortic arch, and the abdominal aorta at the level of the diaphragm. 3 4

The role of transesophageal echocardiography (TEE) in the assessment of the thoracic aorta and acute aortic syndromes is well recognized. 5 6 Although some authors start to recognize the potential applications of TEE in guiding thoracic endovascular repair, 6 7 8 9 its role is not well defined yet. In recent years, the addition of three-dimensional (3D) imaging has improved the anatomical definition of echocardiography and has expanded its applications. 10 Given its additional value, 3D TEE has been commonly used in various cardiac percutaneous interventions 7 10 11 12 and it could equally have additional value in planning or guiding percutaneous aortic procedures.

## Acute Aortic Syndromes



**Video 1** Three-dimensional (3D) transesophageal echocardiography (TEE) image of entry point in aortic dissection.


**Video 2** Three-dimensional (3D) transesophageal echocardiography (TEE) image of pulsatile and small true lumen and large false lumen in a patient with aortic dissection.


Acute aortic syndromes include a spectrum of conditions such as classic aortic dissection (AD), intramural hematoma (IMH), symptomatic penetrating aortic ulcers (PAUs), and traumatic aortic lesions. 13 The prime description is based on both anatomic location of the intimal tear and the degree of propagation of the FL. AD is classified according to extent and entry location by either the Stanford or the DeBakey classification. In a Type I DeBakey's dissection, the intimal tear involves the ascending aorta, and the dissecting lamella extends past the origin of the left subclavian artery, while Type II dissections are confined to the ascending aorta. Type III dissections begin after the origin of the left subclavian artery and extend distally. The fundamental distinction of the Stanford classification is whether the dissection is proximal (Type A) or distal (Type B) to the origin of left subclavian artery. The Stanford system is more widely used because of its simple lettering system and relevance to management. Type A dissection usually requires surgery while Type B dissection can be managed by endovascular methods or medication. 14 Diagnostic confirmation of dissection is the presence of an intimal flap separating true lumen (TL) and FL.

Given the proximity between esophagus and aorta, the diagnostic accuracy of TEE for AD is superior to that of TTE and comparable to contrast-enhanced computed tomography (CT) imaging. The reported sensitivity of 86 to 100% and specificity of 90 to 100% 5 6 further increase when a contrast ultrasound agent is used, as in the case of TTE. 15

**Fig. 1:**
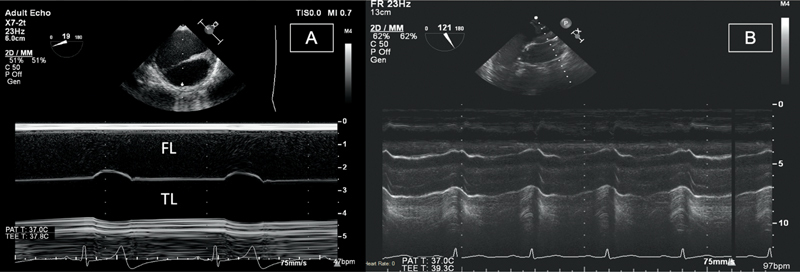
Transesophageal echocardiography and M-mode views of both descending thoracic ( **A** ) and proximal ascending ( **B** ) aorta. Panel A shows false lumen (FL) on top with increased opacification related to slow flow and true lumen (TL) on bottom, expanding in systole. Panel B shows two reverberation artifacts inside the TL of the aorta from adjacent structures.

Diagnostic proof of dissection relies on separating TL from FL; the TL is usually smaller in size with higher flow velocity and pulsatile flow in systole on color Doppler 8 imaging. M-mode is very accurate in demonstrating any expansion of the TL in systole and differentiating artifact from true dissections ( Fig. 1 ). The FL can present with partial thrombosis or total thrombosis at different levels of the aorta.

**Fig. 2:**
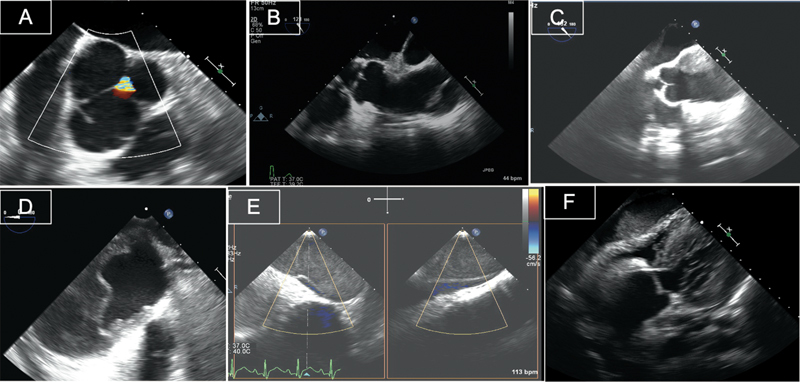
Transesophageal echocardiography of proximal aortic dissection. Mid-esophageal short axis views showing localized dissection of the right coronary cusp, that was missed on computed tomography with central aortic regurgitation ( **A** ), and a dissection flap affecting the aortic root but sparing the origin of the left main coronary artery ( **D** ). Mid-esophageal long axis views with entry tear in the mid-ascending aorta at the level of the right pulmonary artery ( **B** ) and false lumen thrombosis starting at the level of the sinotubular junction ( **C** ). Cross-sectional view and color Doppler of the descending thoracic aorta showing distal dissection with compression of the true lumen ( **E** ). Deep transgastric view demonstrating dense pericardial effusion causing compression of the right ventricle in a patient with Type A dissection; note the dilatation of the aortic root ( **F** ).

**Fig. 3:**
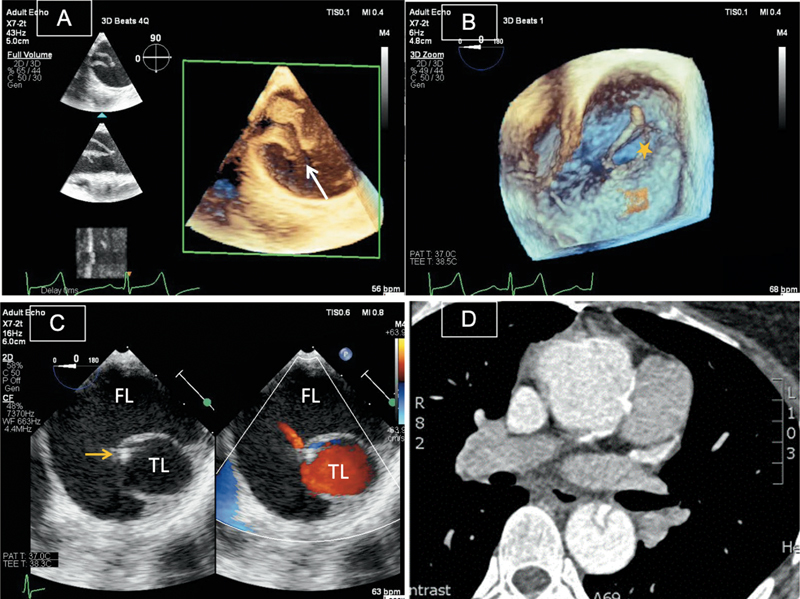
Transesophageal echocardiographic three-dimensional (3D) images showing the entry point (arrow) of the dissection ( **A** ) and the relationship between true lumen (TL) (star) and false lumen (FL) ( **B** ). Two-dimensional (2D) images with and without color Doppler confirming a guidewire (yellow arrow) in the TL and slow flow in the FL ( **C** ). Axial computed tomography image of the same patient, showing TL and FL with good correlation to echocardiography ( **D** ).

During an intervention, advancing and withdrawing the probe can help differentiate when both TL and FL appear initially similar in size. This maneuver permits tracking of each lumen along the aorta, where characteristic features of TL or FL can become more evident, as the presence of spontaneous contrast related to slow flow in the FL or systolic pulsatility in the TL. Attention should be paid at tracking the lumens in complex dissections with spiroid course, when all echocardiographic parameters that help identify each lumen correctly must be reassessed frequently. In addition, TEE is useful to identify proximal retrograde extension of a distal dissection into the aortic root, potential involvement of coronary arteries, dysfunction of the aortic valve, and presence of pericardial effusion ( Fig. 2 ). Pericardial effusion in Type A AD often heralds poor prognosis and suggests rupture of the FL into the pericardium. 16 17 18 Moreover, TEE is even able to identify involvement of celiac trunk and mesenteric arteries, 19 although visualization of visceral and of supra-aortic vessels can be technically challenging. Although the course of the left bronchus 5 generates a blind spot at the level of the distal ascending aorta, 6 new matrix array probes and experienced operators can detect flaps in some cases. The addition of 3D to TEE in the evaluation of dissection further enhances the anatomical delineation of the disease by better identifying the entry size ( Fig. 3 , Videos 1 and 2 ), coronary artery ostia involvement, and the relationship of the dissection flap with surrounding structures, particularly in cases of spiral dissection. 5 20 21

## Thoracic Endovascular Aortic Repair for Type B Aortic Dissection

Endovascular management has become widely accepted as an important treatment modality in complicated and high-risk Type B dissection. 1 14 TEVAR conceptually aims at covering the primary entry and remodeling the aorta by expansion of the TL at the expense of the FL, thereby redirecting flow to the TL—hence correcting distal malperfusion and protecting against long-term aneurysmal degeneration of the distal aorta. 22 Entry points in Type B dissection are usually located just distal to the left subclavian artery and identified as a disruption of the torn media flap with communication of flow between TL and FL. TEVAR is usually performed via femoral artery access with retrograde transarterial advancement of a large bore device (20–26 F) containing a collapsed self-expandable stent-graft, delivered over a stiff guidewire in the TL. As TEVAR requires fluoroscopic guidance during the procedure, 23 TEE as an adjunctive intraoperative imaging technique can provide incremental information before, during, and after the procedure, thus increasing both success and safety of the intervention. 8

### Preprocedural Transesophageal Echocardiography

Preprocedural TEE should focus on both confirming the underlying aortic pathology and providing quantitative approximation of aortic dimensions and flows in TL and FL. Importantly, before starting an endovascular procedure, the proximal ascending aorta needs to be evaluated to exclude its involvement. A general look at cardiac structures and ventricular function is easily performed, with focus on assessment of aortic valve anatomy and function, left and right ventricular function, and presence of intracavity thrombus that could potentially be dislodged during fast right ventricular pacing maneuvers (prior to stent-graft deployment, rapid ventricular pacing is an option to introduce hypotension and avoid downstream endograft displacement). 23 If possible, the supra-aortic vessels should be assessed for presence of aortic plaques that could interfere with apposition of the stent-graft, promoting the occurrence of Type I leaks after TEVAR. 5 Since most TEVAR procedures are performed under general anesthesia, TEE is well tolerated by the patient and does not delay the procedure.

### Periprocedural Transesophageal Echocardiography



**Video 3** Three-dimensional (3D) transesophageal echocardiography (TEE) image of intraprocedural echocardiography, guiding advance of wires inside the true lumen.


**Fig. 4:**
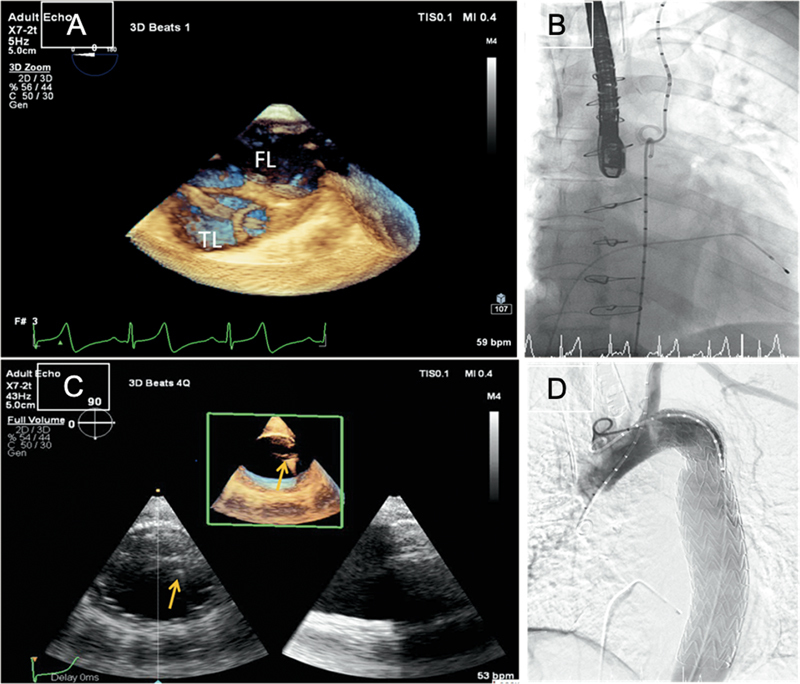
Three-dimensional (3D) transesophageal echocardiography showing a Type B aortic dissection with an embracing pigtail maneuver inside the true lumen ( **A** ) and its corresponding fluoroscopic image ( **B** ). 3D imaging of aorta postdeployment of stent ( **C** ) with wire still in place (yellow arrow) and the corresponding fluoroscopic image ( **D** ).

During the procedure, TEE is instrumental for a correct identification of the TL and accurate placement of wires within it, before any stent-graft deployment. Fluoroscopy alone may fail to ascertain wire positioning and movement along the TL, particularly in cases of spiral course. When a separate pigtail catheter is needed for angiography or the stent-graft is advanced, proper positioning inside the TL is easy to confirm by TEE; thus, every time a wire or a catheter is introduced, TEE can ascertain its position along the aorta during the manipulations by the operators ( Fig. 4 , Video 3 ).

### Postprocedural Transesophageal Echocardiography

Immediately after stent-graft placement, TEE can assess the full expansion of the stent-graft and the successful exclusion of either FL or aneurysmal sac. A “smoke” phenomenon in the FL is a good sign to indicate sealing of an entry tear and initiation of thrombosis. Color Doppler imaging is helpful in detecting residual flow inside the FL or endovascular leaks that would require additional intervention. Persistence of FL perfusion usually indicates failure to close an entry tear or problems with stent-graft apposition, damage to the stent struts, or presence of secondary entry points distal to the stent-graft. Type I endoleaks are located at the proximal edge of the stent and are identified as high jet velocity jets with continuous flow into the FL or aneurysmal sac. In these cases, further balloon dilatation of the stent-graft, a second stent-graft, or even open surgery may be required. The sensitivity of TEE to detect residual leaks in the acute phase (after the stent-graft deployment) is higher than fluoroscopy or CT due to its excellent spatial and temporal resolution and Doppler signal. 8 It is mandatory to rule out retrograde dissection into the aortic arch and proximal ascending aorta. 24 Also, when possible, it should be evaluated whether the supra-aortic branches have been jailed by the stent-graft although the latter is often difficult to see on TEE alone. Other potential complications such as evolution of pericardial effusion, disturbed left ventricular function, or worsening of valve incompetence postpacing should be ruled out before terminating a given case.

## Thoracic Endovascular Aortic Repair for Type A Dissection

The concept of TEVAR for Type A AD has recently been applied in selected cases. Optimistic contrast-enhanced CT-based studies have described up to 50% of patients with proximal dissection possibly amenable to an endovascular approach 25 26 ; moreover, up to 10% of patients with Type A AD are not accepted for surgery due to prohibitively high risk. 9

### Preprocedural Imaging

CT angiography plays a major role in planning the intervention in this highly selected population. Features like the distance between coronary arteries, aortic valve, and neck vessels to entry tears and the length of a safe landing zone are important to be addressed prior to intervention. An entry tear located in the mid-ascending aorta not extending to supra-aortic vessels or aortic root characterizes the most suitable pathology. The absence of an acceptable proximal landing zone is the most common exclusion criterion for endovascular repair, when the necessary distances to coronaries and aortic valve are not confirmed. Identification of entry points in the distal ascending aorta is not always possible on TEE, but TEE can provide indispensable information in the planning of the procedure: such as exclusion of aortic root or coronary artery involvement, assessment of ventricular function, and both degree and mechanism of aortic valve regurgitation. In cases of significant valve dysfunction or entrapment of coronary arteries by the FL, TEVAR would not be an option and surgical repair or medical palliation could be the only choices.

### Intraprocedural Transesophageal Echocardiography

Unlike CT, TEE is able to provide intraoperative guidance and monitoring that can be crucial for procedures in this anatomical location. Similar to a TEVAR procedure for Type B dissection, in a case of endovascular treatment of proximal dissection a guidewire will be inserted retrogradely from a femoral access. For good support during the delivery and the deployment of the stent-graft, the stiff guidewire is placed across the valve into the left ventricle via a pigtail catheter. TEE can confirm the proper positioning of wires and catheters inside the TL and help detect complications such as entrapment of wires or catheters in the mitral subvalvular apparatus, damage to the aortic valve, or pericardial effusion from myocardial perforation.

### Postprocedural Transesophageal Echocardiography

TEE should confirm the correct expansion and positioning of the stent, ongoing FL-induced thrombosis, and lack of complications such as stent migration or coronary artery entrapment.

## Aortic Aneurysms in the Descending Thoracic Aorta

In the treatment of aortic aneurysms in the descending thoracic aorta, TEVAR has demonstrated lower early mortality rates than open surgery. For this reason, TEVAR is the preferred option for treatment when aortic diameter exceeds 55 mm. In recent years, combinations of endovascular stenting at different levels of the aorta, including the arch, and thoracic aorta have evolved. Future directions will include focus on the preservation of flow in the side branches. 27 At present, TEVAR is not recommended for treatment of aortic aneurysms in the ascending thoracic aorta or patients with connective tissue disease, unless as a bridge to surgery. 1

## Intramural Hematoma and Penetrating Aortic Ulcer

IMH and PAU are two entities that can coexist or be identified separately. Each of them can affect the proximal or descending thoracic aorta, although they are more frequently located in the latter. 28 IMH is identified by thickening of the aortic wall (cutoff of 5 mm) caused by bleeding into the media layer without a dissection flap. Despite its low incidence, early diagnosis is important as it can progress to AD, PAU, or even rupture. 29 TEE has the advantage over CT and magnetic resonance imaging to be able to visualize the intimal layer in more detail and detect possible disruptions better than the other techniques. 30 Endovascular interventions in presence of IMH may be advised in presence of uncontrolled pain, extension, periaortic hematoma, or focal intimal disruptions. Primary PAU often occurs in older patients or in presence of atherosclerotic disease and consists of localized ulcerations of the intima layer into the media. If untreated, this pathology can progress to rupture and a bigger size has been suggested to prompt urgent intervention. As with IMH, TEE can provide the diagnosis of PAU by observing a crater-like lesion on an atherosclerotic segment of the aortic wall. 29 Despite the scarce data available to compare TEVAR with surgery in Type B IMH or PAU, TEVAR is currently accepted as the preferred modality because of lower perioperative mortality and less surgical trauma, although decisions should be individualized to the exact characteristics of the patient. 1 31 In Type A IMH or PAU, surgery remains the recommended option to date. 1

## Interventions in Aortic Coarctation

Coarctation of the aorta is a congenital condition that involves increased stiffness of the aortic wall and focal narrowing (sometimes along with hypoplasia of the arch), potentially reducing distal perfusion. Postductal coarctation, with the narrowing occurring distally to the origin of the left subclavian artery, is the most commonly encountered form of coarctation in adults. Percutaneous treatment of native coarctation or re-coarctation has become the firstline approach in current practice and is usually performed with balloon dilatation followed by stenting of the coarctation site. 31 32 Unlike in other congenital heart defects, 11 the role of TEE in the assessment of aortic coarctation is not yet well defined, although it has been used successfully for diagnosis, quantification, and interventional guidance in children. 33 34 35 The reluctance to use TEE may be explained by interference with the angiographic assessment of coarctation and its limitations to image the supra-aortic branches. Conversely, TEE is essential to check for additional intracardiac defects that should always be ruled out, as their association with coarctation is not uncommon.

## Ancillary Aortic Interventions

Ancillary interventional techniques have been proposed to promote FL thrombosis and prevent aneurysmal degeneration. Incomplete or partial FL thrombosis has been associated with increased mortality, as has elevated diastolic pressure in the FL, which increases both wall tension and the risk of distal aneurysmal degeneration or rupture. 36

**Fig. 5:**
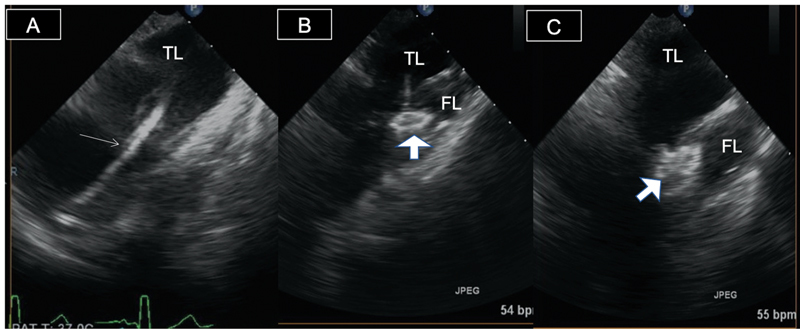
Transesophageal echocardiography demonstrating an endovascular intervention to dissection of the ascending aorta. Wire inside true lumen (TL) (narrow arrow) ( **A** ), advancing the occluder device (thick arrow) from the TL into the false lumen (FL) ( **B** ), and the final result with the occluder device sealing the entry point of the proximal dissection ( **C** ).

**Fig. 6:**
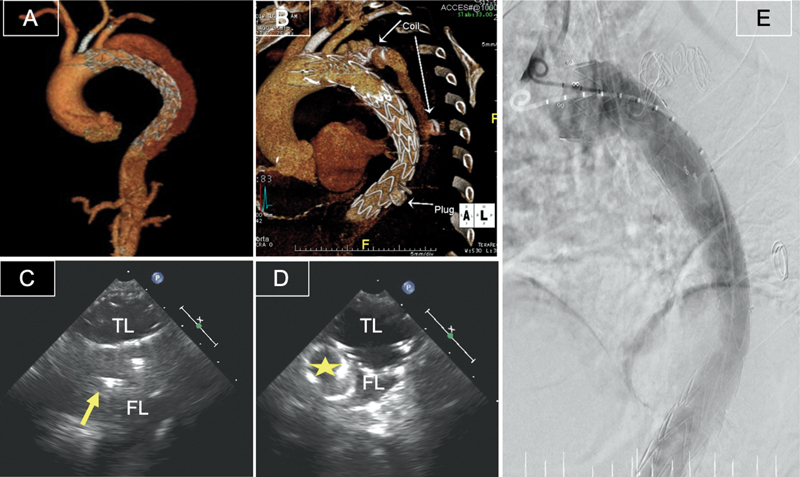
Computed tomography three-dimensional (3D) volume reconstruction of the thoracic aorta in a patient after previous thoracic endovascular repair for Type B dissection, with perfused and expanding false lumen (FL) pre- ( **A** ) and postclosure with coils and vascular plug ( **B** ). Transesophageal echocardiography of the descending thoracic aorta showing coils (yellow arrow) and induced thrombosis of FL ( **C** ) and a vascular plug (star) placed inside the FL ( **D** ). Fluoroscopy postintervention confirming no residual flow in FL ( **E** ). TL, true lumen.

*FL intervention to promote remodeling and thrombosis* (FLIRT) 2 represents a novel conceptual approach after previous dissection or unsatisfactory TEVAR results that can be applied to both the ascending and descending aorta in highly selected cases ( Figs. 5 and 6 ). Closure of entry tears using closure devices, coils, or both can restore single lumen blood flow with promising long-term benefits. 37 TEE guidance is crucial during these procedures for wire positioning inside the FL and correct deposition of coils or vascular plugs. After deployment of occluders and/or coil embolization, color Doppler imaging can detect residual flow in the FL as well as endoleaks that may prompt further intervention.

**Fig. 7:**
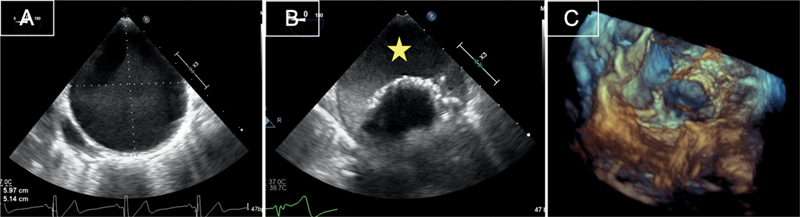
Transesophageal images of aneurysmatic descending thoracic aorta in a patient with Marfan syndrome ( **A** ), managed by endovascular stenting inside the aneurysm with thrombus formation (star) in the excluded aneurysmatic space around the stent graft ( **B** ). Three-dimensional (3D) image showing a stent-graft inside the aneurysm ( **C** ).

*Endovascular repair in hereditary connective tissue disorders* : Patients with Marfan disease have a higher tendency to develop late aortic complications such as retrograde dissection or rupture. Imaging studies based on both ultrasound and CT have tried to investigate the possible related mechanisms. 38 For this reason, TEVAR in this subset of patients remains controversial and current guidelines still discourage the use of endovascular technology for the treatment of descending thoracic aneurysm or in Type B dissection in patients with Marfan syndrome, unless as a bridge to surgery. 1 23 However, TEVAR in patients with previous interposition grafting for Type A AD, would not be at risk of retrograde extension of the dissection 39 and novel endovascular procedures for treatment of a localized aneurysm of aortic branches in Marfan patients have been described with good results. 40 However, further evidence is needed to clarify whether TEVAR could be successfully and safely applied to Type B AD or in aneurysms of the descending aorta in conditions of hereditary connective tissue disease. Even in such patients, the creation of a stent-grafted lumen by percutaneous insertion of an endovascular device anchored proximally and distally to an aneurysmatic segment could induce thrombosis of the excluded aneurysmal sac (or the native dilated aorta), hence reducing both shear stress and wall tension caused by pulsatile flow and thus reducing the risk of further dilatation or rupture ( Fig. 7 ). Nevertheless, additional research in this area is warranted before recommendations are made.

*Percutaneous interventions in the aortic arch* , *including hybrid interventions* , remain a challenging field, although potentially technically feasible and likely beneficial in highly selected cases. The role of TEE in this type of procedure is limited by suboptimal visualization of the origin of all the supra-aortic branches and the interposition of the left bronchus. 5 Nevertheless, TEE can be helpful for the initial assessment of extent of disease and of concomitant findings that may complicate the intervention (i.e., ventricular or valvular dysfunction) and to rule out complications. 35

## Future Directions

*Contrast-enhanced echocardiography* increases the likelihood of detecting an intimal flap and helps in anatomic definition of the aorta to plan individual treatment strategies. 15 Intraprocedural administration of contrast through a pigtail catheter located in the aortic root has been successfully used during FLIRT to facilitate the identification of the entry point and the extent of the disease, and to confirm the results after an intervention. 41 Similarly, it has been found useful to guide TEVAR, 42 with potential benefits using contrast-enhanced ultrasound such as a reduced volume of iodine-based contrast needed, less fluoroscopy time, and increased intraprocedural safety by real-time confirmation of results. However, further experience and studies are needed to establish formal recommendations.

*Intravascular ultrasound* has the advantage to visualize the flap beyond the diaphragm level, down to the abdominal aorta. Previous experience in TEVAR guidance has shown potential to identify multiple entries and FL thrombosis after an intervention, but the identification of endoleak was less accurate than with TEE. In addition, simultaneous imaging and delineation of intracardiac anatomy is not possible with intravascular ultrasound. 8

*3D printing models* have emerged in recent years as an additional tool to help understand complex underlying anatomy. As for aortic diseases, initial studies of aortic aneurysm and dissection have shown encouraging results, and further development of this supportive technology may be helpful in the teaching and planning of percutaneous aortic procedures. 43

*Fusion imaging using a combination of CT reconstructions and fluoroscopy* has shown promising results in terms of reduction of the amount of contrast and radiation exposure. 44 Whether TEE could further improve fusion imaging or safety of any endovascular procedure needs further exploration.

## Limitations

TEE is a semi-invasive procedure and may therefore carry a small risk of procedural complications. 45 46 Patients with absolute contraindications such as perforated viscus, esophageal stricture, tumor, perforation, laceration, or diverticulum, or active upper gastrointestinal bleed, should not undergo TEE monitoring during any intervention ( Table 1 ). 46 The use of TEE will require the need for sedation or even general anesthesia, under which endovascular procedures are conducted anyways. TEE is a highly operator-dependent imaging modality that requires advanced pathoanatomic knowledge, operational skills, and understanding of the interventional aortic procedure itself. As previously mentioned, even in experienced hands visualization of the distal ascending aorta, proximal arch, and supra-aortic branches is often limited. 3 Flawless communication with the interventional team is also required during any of the above procedures.

**Table 1:** Contraindications to transesophageal echocardiography recommended by the American Society of Echocardiography and the Society of Cardiovascular Anesthesiologists 46

Absolute contraindications	Relative contraindications
Perforated viscus	History of radiation to neck and mediastinum
Esophageal stricture	Restriction to neck mobility
Esophageal tumor	History of upper gastrointestinal bleed or surgery
Esophageal perforation, laceration	History of dysphagia
Esophageal diverticulum	Barrett's esophagus or esophageal varices
Active upper gastrointestinal bleed	Active esophagitis or peptic ulcer
	Symptomatic hiatal hernia
	Coagulopathy, thrombocytopenia

## Conclusions

Endovascular interventions on the aorta are an attractive alternative to surgery, with expanding applications in the aging population, some of which are already established as preferable options over open surgery under certain circumstances. 1 TEE provides key anatomical information about the aorta and can be instrumental for real-time intraprocedural guidance and immediate detection of complications. Future studies should confirm whether TEE in fact reduces fluoroscopy time or the amount of contrast dye used. Advanced training in TEE and close collaboration with the interventional team is required to add value to any endovascular procedure applied to the aorta.
